# Department of Practice of Medicine

**Published:** 1893-04

**Authors:** Allen J. Smith

**Affiliations:** Medical Department University of Texas, Galveston


### DEPARTMENT OF PRACTICE OF MEDICINE.

Conducted by Professor Allen J. Smith, A. M , M. D., Medical Department University of Texas, Galveston.

DILATATION OF THE COLON IN YOUNG CHILDREN.—Osler (*Archives of Pediatrics*, February, 1893) records the instances of distention of the large bowel in children from coprostasis. One of these children, a colored boy of ten years of age, whose previous history was indefinite, presented a highly distended abdomen, measuring sixty-three centimeters in circumference, the body weight being 47.5 pounds. The lad was in a general way constipated, but not remarkably so, and all efforts to diminish the abdominal distention by means of purgation had failed. His general condition was such that, in view of failure to locate any cause for the condition, an exploratory laparotomy was performed and an extraordinarily enlarged colon was encountered, the greatest distension in the region of the sigmoid flexure, where the gut was forty-five centimeters in circumference. No stricture of the rectum was found, but in the very unpromising condition of the child it was thought best to establish an artificial anus, which was done, large quantities of yellowish fecal matter and gas escaping. The wisdom of this apparently extreme measure was seen in the rapid improvement of the child, and his quick increase in weight. A second child is mentioned. seven months old, very constipated, and with abdomen much distended, save after efforts of the mother to cause movements of the bowels, accomplished by injecting a few ounces of water and a few hours afterwards passing a rectal catheter, bringing away quantities of feces and gas. There could be found no stricture of the rectum. When born the child was apparently healthy, but from the outset the napkins were not soiled and the abdomen became so swollen and tense that finally the physician introduced a rectal catheter, when the first tarry stool was evacuated. Since birth there had been natural movements of the bowel on only two or three occasions.

These cases suggest that often abdominal enlargements are associated with an early condition, in which, without demonstrable tightness of the sphincter or stricture of the rectum, the bowel is unable to empty itself. The regulation of diet and the relief of the bowel by irrigation would seem to be the special indications in the treatment.

---

THE PERIOD OF INCUBATION OF CHICKENPOX.—Eyre (*British Medical Journal*, 1892, vol. 2, page 1430; *Archives of Pediatrics*, February, 1893) relates the following: On November 18, Mrs. M., of Hampstead, visited her sister, Mrs. O., residing at Streatham, taking with her her little boy. They stayed three hours at Mrs. O.'s house. Soon after they left Mrs. O. found one of her children with a number of pimples upon her body, and the physician who saw the child the next day diagnosed the case as one of chickenpox. Mrs. M.'s child was taken with the same decease, on December 2d, and the rash appeared the next day.

---

PERIOD OF INCUBATION OF MUMPS.—Jessop (*British Medical Journal*, June 4, 1892) reports the case of a boy who was brought in contact with a person recovering from mumps on March 17; on April 19, having been in perfectly good health in the interval, he developed a typical attack of mumps. No other exposure was known. On the day of the onset of the disease, April 19, his two sisters kissed him. They were then removed and did not see him again. Twenty-one days later, May 16. they also were attacked with mumps. This places the period of incubation in these cases definitely at three weeks. All the children were quite well during the interval between the exposure and the development of the disease.—*N. Y. Med. Journal,* February 11, 1893.

---

PERIOD OF INCUBATION OF DIPHTHERIA.—M. B. Dwight (*Medical Age*, February 25, 1893) states that from the observation of about twenty cases of diphtheria during an epidemic some years since he was able to place the period of incubation at forty-eight hours. The narration of his cases is of interest.

In July, I884, two children, a boy and his sister, aged respectively nine and eleven years, inmates of a soldier's orphan home, made a visit to their relatives living in Jersey Shore, Pa. They first visited a family in which there were three children, arriving late at night on July 24. Late on the evening of July 26 two of the childen visited were attacked with sore throat and fever. In the meantime the orphan children had gone several miles further up the valley, known as Pine Creek valley. Two days later Dr. Dwight was called to a family in this district, visited by the same children, in which there were seven children, one of whom had diphtheria. The orphans had separated in their journey up the valley, and while the girl had visited at the last named family the boy had remained at a house half a mile away. After night he rejoined his sister. Of the two children at the house he had visited, one, with whom he had slept, became infected with the diptheria.

Thence the orphans retraced their steps back to Jersey Shore, a distance of two miles or more. On their way they visited a house for about two hours. There were several children here and from his previous experience the physician predicted in the family the occurrence of diphtheria within forty-eight hours. The result justified his prophesy; two, the mother and a child becoming ill two days after the visit. The orphans had gone before Dr. Dwight came up to the house, and had gone over a range of mountains into Nippenose Valley, a distance of fifteen miles. Dr. Dwight followed them the next day and examined them. They denied having had diphtheria, but their throats had a suspicious appearance. Dr. Dwight again predicted diphtheria in this last family and two days later his prediction was verified.

---

THE MEDICAL DEPARTMENT OF WESTERN RESERVE UNIVERSITY at Cleveland, Ohio, received from Mr. J. L. Wood, as a Christmas gift, the sum of $125,000. Of this $100,000 are to be devoted to the endowment of five chairs to be known as the John L. Wood's Professorships of Histology, Physiology, Chemistry, Anatomy and Pathological Anatomy. The other $25,000 form a reserve fund, the income from which shall be devoted to repairs required upon the building. This medical building was erected by the same gentleman recently at a cost of $200,000; and he also contributed to the Emily Wood's Endowment Fund $50,000.—*Cleveland Medical Gazette*, Dec., 1892.

---

"Are there too many doctors?" asks an exchange.

"No, there are not half enough; but there are too many men pretending to be doctors, who are not.—*Texas Siftings*.

---

THE United States has one drug store to every three doctors.—*Ex*.

---

PASTEUR celebrated his seventieth birthday on the 27th of December last, by a reception held in the Sorbonne. The President of the Republic, all the high government officials, the members of the Academy, and the leading scientists were present to congratulate him. Addresses were presented from England, Russia, Germany, and many other countries.

